# Exploratory Genome-Wide Association Study of Grapefruit Intake and Its Potential Link to Obesity Risk in US Cohorts

**DOI:** 10.3390/nu18091319

**Published:** 2026-04-22

**Authors:** Ji Hyun Bae, Hyunju Kang

**Affiliations:** 1 Department of Food Science and Nutrition, Keimyung University, Daegu 42601, Republic of Korea; jhb@kmu.ac.kr; 2 School of Food Science and Biotechnology, Kyungpook National University, Daegu 41566, Republic of Korea; 3 Center for Food and Nutritional Genomics Research, Kyungpook National University, Daegu 41566, Republic of Korea

**Keywords:** grapefruit intake, genome-wide association study, obesity risk, precision nutrition

## Abstract

**Background/Objectives**: Understanding the genetic basis of food consumption is a key step toward precision nutrition, viewed as a long-term future perspective. This study aimed to investigate genetic variants associated with grapefruit (*Citrus paradisi*) intake and to evaluate their potential relationship with obesity risk. **Methods**: A genome-wide association study (GWAS) was conducted on 19,653 European-ancestry participants from two prospective cohorts, the Nurses’ Health Study (NHS) and the Health Professionals Follow-Up Study (HPFS). We employed a functional annotation strategy to select a suggestive locus for follow-up analysis, and computationally derived molecular docking simulations explored a plausible functional link between grapefruit’s bioactive compounds and the candidate gene product. **Results**: Although falling short of the conventional threshold for genome-wide significance, a suggestive locus was prioritized on chromosome 14, with the lead single nucleotide polymorphism (SNP), rs2124 (*p* < 5 × 10^−6^), located within the metabolic gene *ADCK1* (aarF domain containing kinase 1). Molecular docking simulations supported a plausible mechanistic hypothesis, indicating that key bioactive compounds in grapefruit could bind with high affinity to the ADCK1 protein. Consistent with the GWAS finding, individuals with the CC genotype reported lower mean grapefruit intake. This genotype was also associated with other lifestyle factors, notably, lower physical activity in women. In age- and multivariate-adjusted models, the CC genotype was associated with a modestly increased risk of incident obesity in females, but not in males. **Conclusions**: Our exploratory findings suggest a prioritized candidate locus associated with grapefruit intake, and its link to obesity risk may be mediated by the metabolic gene *ADCK1*. However, given the lack of genome-wide significance and independent replication, these findings should be considered preliminary and exploratory. These hypothesis-generating results support the integration of genetics and dietary habits, warranting further mechanistic validation.

## 1. Introduction

Grapefruit (*Citrus paradisi*) is consumed widely in today’s health-conscious populations for its perceived protective effects against cardiovascular diseases and cancers [1]. Historically, grapefruit has been incorporated into calorie-restricted dietary interventions, most notably the “grapefruit diet”, to evaluate its potential effects on weight loss and metabolic health [2]. Grapefruit’s nutritional value is largely attributed to its rich composition of bioactive components, including flavonoids, carotenoids, and dietary fibers, which are suggested to offer benefits such as improved lipid metabolism, glycemic control, and effective weight management [3,4]. However, taste perception variability can significantly influence individual grapefruit consumption patterns, potentially altering its associated health benefits and providing a rationale for genetic investigation.

Dietary behaviors and food preferences significantly influence nutritional intake, health outcomes, and chronic disease risk, including obesity [5]. In the United States, obesity prevalence has steadily increased, with approximately 40.3% of adults classified as obese according to recent Centers for Disease Control and Prevention (CDC) reports [6]. Addressing modifiable dietary factors, including personalized food preferences, represents a crucial public health strategy to mitigate obesity and related metabolic disorders [7]. While many genes have been linked to obesity susceptibility, such as *MCM6*, *MAPRE3*, and *UBXN4*, which are also independently associated with metabolic disorders [8], the specific genetic links between the consumption of particular foods and obesity are less defined. Although obesity is known to be highly heritable and genetically complex [9], its potential associations with grapefruit consumption have not been thoroughly investigated. Investigating these associations is a necessary step to clarify how genetics, diet, and metabolic health interact.

Relationships between genetic variations and taste preference underpin the concept that taste perception can influence individual food choices and dietary habits [10]. Genome-wide association studies (GWAS) have successfully identified genetic variants linked to the consumption of foods characterized by basic tastes, such as sweet, bitter, and umami [11,12,13]. We previously observed a relationship between sweet and salty taste preferences and obesity risk using GWAS [13,14]. Nevertheless, information on the genetic determinants of the intake of foods with more complex flavor profiles, particularly for nutritionally important fruits like grapefruit, remains limited. Notably, while previous candidate gene studies have linked bitter taste receptor genes, such as *TAS2R38* to grapefruit bitterness perception [15,16], a comprehensive, genome-wide search for suggestive loci associated with actual grapefruit consumption is lacking. To advance personalized nutrition strategies and inform the development of targeted functional foods [17], systematic analyses of these genetic determinants are essential.

Thus, the present study aimed to conduct an exploratory, hypothesis-generating GWAS to investigate genetic loci associated with grapefruit intake, and to subsequently evaluate whether the prioritized candidate loci from this GWAS showed any association with incident obesity risk, using data from the Nurses’ Health Study (NHS) and the Health Professionals Follow-up Study (HPFS). As a long-term future perspective, by integrating genetic insights into dietary behavior and metabolic outcomes, this research aims to contribute to the field of precision nutrition and explore potential pathways for tailored dietary interventions aimed at obesity prevention.

## 2. Materials and Methods

### 2.1. Study Participants

This study utilized data from two well-established prospective cohort studies: the Nurses’ Health Study (NHS), initiated in 1976 with 121,701 female registered nurses aged 30 to 55 years, and the Health Professionals Follow-up Study (HPFS), launched in 1986 with 51,529 male health professionals aged 40 to 75 years [18,19,20]. At baseline, participants completed detailed questionnaires on medical history and lifestyle factors, and have since been followed biennially. Semiquantitative food-frequency questionnaires (FFQs) have been administered every 2–4 years for the NHS since 1980 and every 4 years for HPFS since its inception in 1986.

The present analysis included participants with available genetic data from 1986 to 2014 (18,499 women and 10,878 men). From this group, individuals with pre-existing diabetes, cancer, or a body mass index (BMI) greater than 30 kg/m^2^ at baseline were excluded. Additional exclusions were made for missing or implausible dietary data. After applying all clinical exclusions and genomic quality control steps, the final analytic sample for the GWAS consisted of 12,098 women and 7555 men (total *n* = 19,653) [21,22]. Ethical approval was obtained from the institutional review boards of Keimyung University (approval no. 40525-202110-BR-066-01), Brigham and Women’s Hospital, and the Harvard T.H. Chan School of Public Health. Informed consent was implied by participants’ completion and return of the baseline questionnaire.

### 2.2. Assessment of Obesity and Covariates

The primary outcome of this study was incident obesity, defined as a BMI exceeding 30 kg/m^2^. BMI was calculated from self-reported height and weight, which were collected via biennial questionnaires. Information on potential confounding variables was collected at baseline and updated biennially. These included demographic factors (age, ethnicity), lifestyle habits (smoking status, physical activity), and dietary components from the FFQs (total energy intake, trans fat intake, and alcohol consumption). Physical activity was quantified as the average weekly time spent on activities such as walking, running, and bicycling, and was expressed in metabolic equivalent of task (MET) hours per week [18,19].

### 2.3. Dietary Assessment and Bitter Taste Preference Phenotype Definition

Food intake frequencies were computed from the FFQ data and categorized into nine groups: never/almost never, 1–3 times/month, once/week, 2–4 times/week, 5–6 times/week, once a day, 2–3 times/day, 4–5 times/day, and >6 times/day. From this information, the cumulative average intake of grapefruit was calculated. To create a dichotomous phenotype for GWAS, participants were categorized based on the distribution of their intake. This comparison of extreme quartiles is a standard approach used to maximize statistical power and enhance the detection of genetic signals associated with a continuous trait. The ‘low intake’ group was defined as those in the lowest quartile (25th percentile) of cumulative average intake, while the ‘high intake’ group comprised those in the highest quartile (75th percentile).

### 2.4. DNA Extraction and Genotyping

Biological samples were collected from a subset of participants: in NHS, 32,826 blood and 29,684 cheek cell samples; in HPFS, 18,159 blood and 13,956 cheek cell samples. DNA was extracted from white blood cells or buccal cells for genotyping. As described previously [23], genotyping was conducted at the Dana Farber/Harvard Cancer Center High-Throughput Polymorphism Detection Core and several other laboratories using OncoArray (Illumina, Inc., San Diego, CA, USA), and the Affymetrix 6.0 array (Affymetrix, Santa Clara, CA, USA). To harmonize these heterogeneous genotyping platforms and maximize statistical power, genotype imputation was necessary. We applied stringent quality control (QC) procedures at both the sample and variant levels prior to imputation. We excluded samples with a call rate below 95% (which served as our primary metric to ensure high DNA quality) and removed closely related individuals to ensure sample independence. At the variant level, we excluded single nucleotide polymorphisms (SNPs) with a minor allele frequency < 1% or those deviating from Hardy–Weinberg equilibrium (HWE; *p* < 1 × 10^−6^). Pre-phasing and imputation were performed using the Haplotype Reference Consortium (HRC) as the reference panel [24]. To prevent bias from poorly imputed variants, we strictly excluded any SNPs with an imputation quality score (r^2^) < 0.5 in the post-imputation QC step.

### 2.5. Functional Annotation and Gene Mapping

Since no SNPs reached the conventional genome-wide significance threshold (*p* < 5 × 10^−8^), our strategy shifted from discovery to systematic prioritization. We focused on functionally annotating all suggestive loci (defined as *p* < 1.0 × 10^−5^) to explore the most biologically plausible candidates based on a convergence of functional evidence, rather than *p*-value alone. To explore genomic risk loci and functionally annotate relevant variants, we employed the Functional Mapping and Annotation (FUMA) platform. Risk loci were defined by grouping lead SNPs that were in linkage disequilibrium and located within 250 kb of each other [25,26]. To predict their functional consequences, SNPs within these loci were annotated with Combined Annotation Dependent Depletion (CADD) scores [27] and RegulomeDB scores [28]. Suggestive loci were further mapped to genes in FUMA as follows: positional mapping, which locates the protein-coding gene closest to a given SNP (within a 10 kb window, using GRCh37/hg19 as the reference genome), and expression quantitative trait locus (eQTL) mapping, which utilizes data from the GTEx repository [29] to link SNPs to genes based on significant eQTL associations. Additionally, the functional consequences of non-coding SNPs in haplotype blocks were analyzed using HaploReg v4.1 and the RefSeq database [30].

### 2.6. Statistical Analyses

Participants were followed from baseline (1986 for HPFS, 1980 for NHS) until one of the following events occurred: development of obesity (BMI > 30 kg/m^2^), death, or the end of follow-up in 2014. Individuals with a history of cancer or diabetes at baseline were excluded from this analysis. Genome-wide association analyses were performed using PLINK version 1.9 with a logistic regression model, assuming an additive genetic effect. Within the PLINK analysis, the model was adjusted for age, sex, and the first 10 principal components as covariates to control for population stratification. To assess potential systemic bias, we generated a Quantile-Quantile (Q-Q) plot and calculated the genomic inflation factor (λ). The METAL software (version 2011-03-25; Center for Statistical Genetics, University of Michigan, Ann Arbor, MI, USA) was used for meta-analysis across the two cohorts, and heterogeneity was evaluated using Cochran’s Q test [31,32,33].

To examine the association between lead SNPs and incident obesity, we used Cox proportional hazards modeling with time-varying covariates to estimate age-adjusted and multivariable-adjusted hazard ratios (HRs) and 95% confidence intervals (CIs). We constructed a multivariable model (Model 2) to clearly disentangle the effects of genetic variation from lifestyle and dietary factors. Specifically, Model 2 was adjusted for physical activity (MET-hours/week) to address lifestyle differences and for sweetened beverage intake to strictly isolate the genetic association from the confounding effects of added sugars. Additional covariates included age, smoking status, alcohol consumption, total fiber intake, trans fat intake, and total energy intake. The adjusted HRs for both cohorts were pooled using a fixed-effect meta-analysis conducted with either SAS software (version 9.3; SAS Institute Inc., Cary, NC, USA) or R software (version 4.0.0; R Foundation for Statistical Computing, Vienna, Austria). Additionally, rs713598, a well-established variant in the bitter taste receptor gene *TAS2R38*, was included in the secondary analysis as a positive control for bitter food preference. All *p*-values were two-sided, with a threshold of *p* < 0.05 defining statistical significance. Given the exploratory nature of the secondary phenotype analyses, nominal *p*-values are reported without multiple testing correction (e.g., Bonferroni or FDR).

### 2.7. Plausibility Testing via Molecular Docking Simulation

The 3D structures of human ADCK1 (AlphaFold ID: AF-Q86TW2-F1-v4), naringenin (PubChem ID: 932), and bergamottin (PubChem ID: 5471349) were retrieved from their respective databases. Using AutoDockTools (ADT; version 1.5.7, The Scripps Research Institute, La Jolla, CA, USA), the structures were prepared by adding polar hydrogens, assigning Kollman charges, and converting them to the PDBQT format. Molecular docking was performed with AutoDock Vina (version 1.2.5, The Scripps Research Institute, La Jolla, CA, USA), targeting the protein’s ATP-binding site. A grid box of 20 × 30 × 30 Å^3^ (center x, y, z: −13.0, 11.0, 7.0) was utilized with an exhaustiveness of 9. Final binding poses and interactions were visualized using PyMOL (version 3.0; Schrödinger, New York, NY, USA), as previously described [34]. The reliability of the docking results was supported by the consistency of binding poses and favorable scoring parameters within the AutoDock Vina framework.

## 3. Results

### 3.1. Genome-Wide and Locus-Specific Associations with Grapefruit Intake

**Figure 1:**
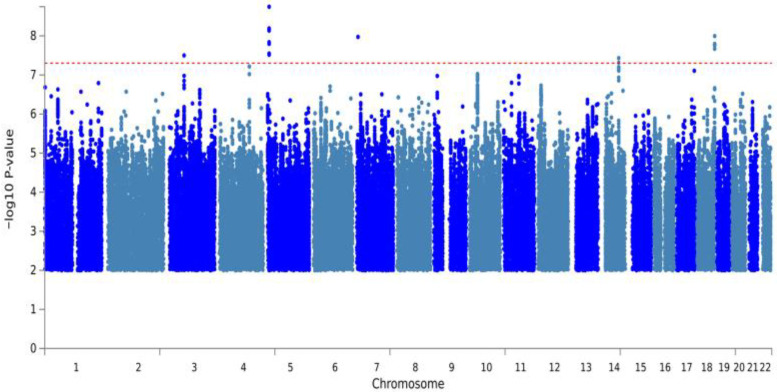
Manhattan plot for the grapefruit intake phenotype from GWAS. Each dot represents a SNP, plotted by its chromosomal position (x-axis) and its −log_10_ *p*-values (y-axis). The red dashed line denotes the conventional genome-wide significance threshold (*p* = 5 × 10^−8^). The plot displays several suggestive loci, with the strongest signal observed on chromosome 5.

To explore genetic variants associated with grapefruit intake, a GWAS was conducted. The analysis showed no evidence of systematic bias from population stratification, as confirmed by visual inspection of the Quantile-Quantile (Q-Q) plot. Figure 1 shows a Manhattan plot in which no SNPs reached the conventional genome-wide significance threshold (*p* = 5 × 10^−8^). We then characterized the most significant genetic signals suggested by GWAS. Specifically, the eight top-ranking SNPs showing the strongest associations with grapefruit taste preference were selected based on a suggestive significance threshold (*p* < 1.0 × 10^−5^), as previously described [35] and detailed in Table 1.

The SNP with the lowest *p*-value was rs6555388 at the *CTD-2324F15.2* locus on chromosome 5 (*p* = 1.80 × 10^−7^) with an OR of 1.22 (95% CI: 1.13–1.32), but its Combined Annotation Dependent Depletion (CADD) was very low, suggesting a lower likelihood of functional impact [36]. Thus, in accordance with our prioritization strategy described in Materials and Methods, we focused on the next most significant signal, rs2124 on chromosome 14 (*p* = 3.69 × 10^−6^). This SNP (Effect Allele = C) had an OR of 0.85 (95% CI: 0.79–0.91), indicating that C-allele carriers were less likely to be in the high-intake group. This decision to prioritize rs2124 was based on a convergence of stronger functional evidence: rs2124 not only exhibited a higher CADD score but was also supported by RegulomeDB scores and expression quantitative trait locus (eQTL) evidence in metabolically relevant tissues, such as pancreas, adipose, and hypothalamus. Based on this systematic, data-driven prioritization, rs2124 was selected as the lead candidate for functional follow-up [13]. This SNP is an intronic variant located within the aarF domain containing kinase 1 (*ADCK1*) gene, a key regulator of energy metabolism.

**Figure 2:**
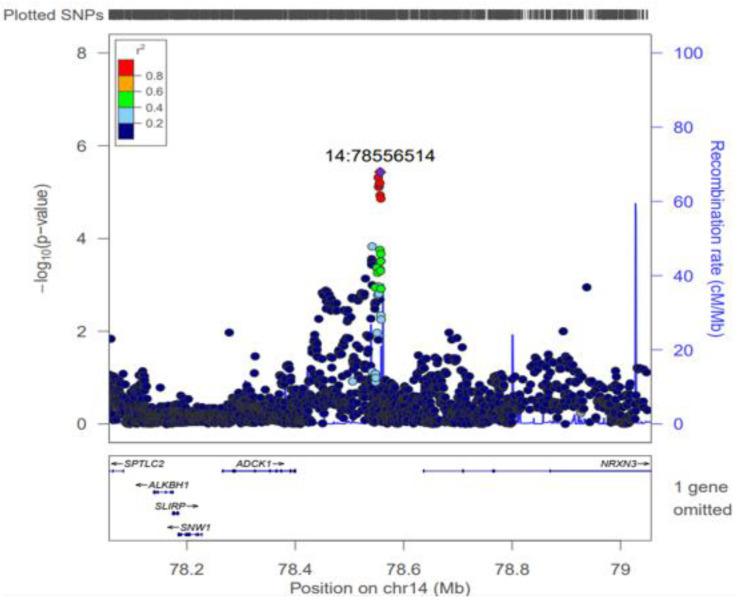
Regional association plot for the suggestive locus on chromosome 14. SNPs are plotted by their genomic position (x-axis) against their −log_10_ *p*-value (y-axis). The prioritized lead SNP, rs2124 (purple diamond), is shown at position 14:78556514. Surrounding SNPs are colored according to their linkage disequilibrium (r^2^) with rs2124. Gene annotations for this region, including *SPTLC2*, *ALKBH1*, *SLIRP*, *SNW1*, *ADCK1*, and *NRXN3*, are shown at the bottom. Arrows on the gene annotations indicate the direction of transcription.

To visualize this prioritized region, a regional association analysis was conducted for the locus on chromosome 14. Figure 2 shows the regional association plot spanning 78.1 to 79.0 Mb, highlighting the prioritized lead SNP at position 14:78556514, with surrounding SNPs colored by their linkage disequilibrium (LD). Gene annotations in the region include serine palmitoyltransferase long chain base subunit 2 (*SPTLC2*), histone H2A dioxygenase (*ALKBH1*), SRA stem-loop interacting RNA binding protein (*SLIRP*), SNW domain containing 1 (*SNW1*), *ADCK1*, and neurexin 3 (*NRXN3*). Although *NRXN3* is a known reward-related gene, the prioritized SNP rs2124 and its surrounding cluster of highly correlated SNPs (r^2^ ≥ 0.8) exhibited strong eQTL signals strictly specific to *ADCK1* in metabolically relevant tissues. As shown in the plot, this cluster is located within the *ADCK1* gene, supporting the hypothesis that *ADCK1* is one of several plausible candidate genes underlying this suggestive association signal.

### 3.2. Molecular Docking Simulations of Grapefruit Compounds with ADCK1

To investigate a potential functional link between the candidate gene, *ADCK1*, and grapefruit-specific compounds, we performed in silico molecular docking simulations. We selected two primary bioactive compounds responsible for grapefruit’s characteristic flavor profile, including naringenin and bergamottin. Naringenin, a flavanone, was specifically chosen because it represents the biologically active form absorbed by the human body [37,38]. While its glycoside precursor, naringin, produces the fruit’s bitter taste, it is metabolized into the aglycone naringenin by gut microbiota prior to absorption [39]. Bergamottin, a furanocoumarin, also contributes to the fruit’s complex sensory experience, including elements of bitterness and aroma [40].

**Figure 3:**
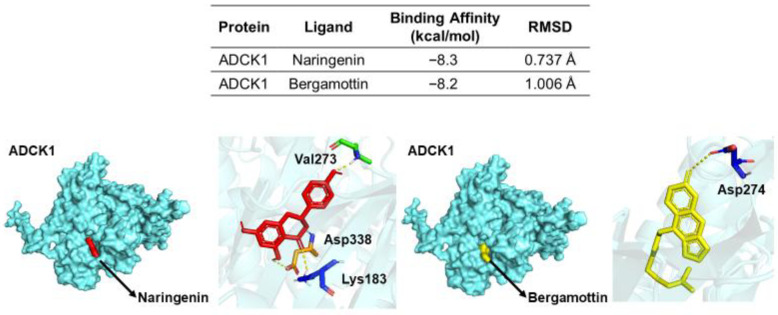
Molecular docking analysis of grapefruit bioactive compounds with the ADCK1 protein. The table displays the calculated binding affinity (kcal/mol) and root-mean-square deviation (RMSD, Å) for naringenin and bergamottin. The visualizations below show the binding poses of naringenin (red) and bergamottin (yellow) within the binding site of the ADCK1 protein (cyan surface). Inset images provide a detailed view of the key interacting amino acid residues (shown as sticks) within the binding pocket.

The docking results revealed that both compounds displayed strong, stable binding within the ATP-binding pocket of the ADCK1 protein (Figure 3). Naringenin exhibited a binding affinity of −8.3 kcal/mol, and bergamottin showed an affinity of −8.2 kcal/mol, indicating favorable interactions. Of particular significance, naringenin formed key hydrogen bonds with Lys183, a residue known to be critical for the catalytic function of the kinase domain [41,42]. Additional interactions, such as those with Asp338 and Val273, further stabilized the compounds’ positions. Bergamottin also formed a key hydrogen bond with Asp274 within the binding pocket.

Crucially, the predicted binding of these bioactive compounds directly within the ATP-binding pocket suggests a computationally derived, speculative mechanism of competitive inhibition, whereby they could modulate the kinase activity of ADCK1 by physically blocking the binding of ATP. These findings provide a plausible molecular mechanism supporting the GWAS association, suggesting that genetic variations within *ADCK1* could alter the structure of its binding pocket, thereby modifying its affinity for grapefruit taste compounds and ultimately influencing an individual’s grapefruit intake.

Functional annotation through CADD and RegulomeDB scores, together with expression quantitative trait locus (eQTL) evidence in relevant tissues (e.g., pancreas, adipose, hypothalamus), further supported the potential regulatory roles of these variants. Based on this functional evidence, the prioritized SNP rs2124 was chosen as the lead SNP. This approach highlights that the genetic effects on dietary behavior can be subtle and distributed across a broad genetic landscape. Overall, the findings suggest a prioritized candidate locus on chromosome 14, warranting further investigation [13].

### 3.3. Characteristics of Study Participants by rs2124 Genotype

To explore the phenotypic characteristics associated with the prioritized SNP, we examined demographic, lifestyle, and dietary factors across its genotypes, with results presented in Table 2. While most baseline characteristics, including age, BMI, smoking status, alcohol intake, physical activity, and total energy consumption, were generally balanced, several notable and often cohort-specific associations emerged. In the male cohort (HPFS), carriers of the C allele (CT and CC genotypes) showed similar patterns when compared to the TT reference group in that they were more likely to be never-smokers, reported higher levels of physical activity, and had a lower intake of alcohol, fruit, vegetables, coffee, tea, kale, and grapefruit. The only exception was for CT carriers, who reported a higher intake of sweetened beverages compared to the TT or CC groups.

The associations in the female cohort (NHS), however, were more complex and genotype-specific. Homozygous (CC) carriers were more likely to be never-smokers and consumed less alcohol, fruit, vegetables, coffee, tea, and sweetened beverages. Consistent with the GWAS findings, they also reported a significantly lower intake of grapefruit. However, in a pattern opposite to that of males, these women reported lower levels of physical activity. In contrast, heterozygous (CT) women showed yet another distinct pattern. They consumed more vegetables, coffee, sweetened beverages, and kale, but less alcohol, compared to the other genotypes. Notably, their grapefruit consumption did not differ significantly.

### 3.4. Associations of the Prioritized SNP with Obesity Risk

We next performed a secondary analysis to explore whether the prioritized SNP, rs2124, was associated with the risk of incident obesity. Table 3 presents hazard ratios (HRs) and 95% confidence intervals from age-adjusted (Model 1) and multivariate-adjusted (Model 2) Cox regression models. The results revealed a notable cohort-specific association. In the female cohort (NHS), the CC genotype was associated with a modestly increased risk of obesity in the fully adjusted model (HR: 1.18; 95% CI: 1.04–1.33; *p* = 0.011). In contrast, no significant association was observed in the male cohort (HPFS) for either the heterozygotes (CT) or homozygotes (CC). In a pooled meta-analysis, the CC genotype showed a nominally significant, hypothesis-generating signal with obesity risk (*p* = 0.043). However, this result appears to be driven by the effect observed in the female cohort. Because the NHS and HPFS are distinct cohorts with differing baseline lifestyle characteristics, we cannot definitively attribute this divergence to a biological cohort-specific difference without formal interaction testing.

Additionally, as a positive control, the well-established bitter taste receptor variant rs713598 (*TAS2R38*) also demonstrated significant associations with obesity risk in the female cohort (e.g., HR for GG vs. CC: 1.141; *p* = 0.038), further supporting the relevance of taste-related genetic variation to metabolic outcomes.

## 4. Discussion

In this exploratory genome-wide association study, we investigated genetic loci significantly associated with grapefruit intake. While no variants reached the conventional genome-wide significance threshold, we prioritized a suggestive locus on chromosome 14 near the metabolically relevant gene, *ADCK1*. This decision was supported by functional evidence from CADD scores and molecular docking simulations, which suggested that key bioactive compounds in grapefruit bind with high affinity to the ADCK1 protein. Phenotypic analysis revealed that the prioritized SNP, rs2124, was also linked to lifestyle habits in a cohort-specific manner, which was most apparent in our secondary analysis of obesity risk, where the CC genotype was associated with increased obesity risk in females, but not in males. Given the central role of taste in shaping dietary behavior [44], these findings generate a hypothesis linking a candidate gene involved in energy metabolism (*ADCK1*) to both a specific dietary behavior (grapefruit consumption) and a metabolic outcome (obesity risk).

This association between the prioritized SNP, rs2124, and obesity risk was notably prominent in the female cohort. In males, C-allele carriers (CT or CC) showed no increased risk of obesity, which may be explained by their more favorable lifestyle profile, including higher rates of physical activity. Conversely, the increased obesity risk observed in female CC homozygotes aligns with their significantly lower reported physical activity, a pattern directly opposite to that seen in males. Although CC homozygote females exhibited lower physical activity, the association with obesity risk remained significant even after rigorous multivariate adjustments for physical activity and sweetened beverage intake. This indicates that the genetic effect operates independently of these behavioral differences, suggesting a potential metabolic consequence, possibly via ADCK1-mediated energy homeostasis. However, as with any observational study, we cannot entirely rule out the possibility of residual confounding. Furthermore, there remains uncertainty in the exact causal direction, specifically, whether the genetic variant primarily influences sensory preference directly, or if post-ingestive metabolic adaptations shape dietary behavior. Consequently, it is challenging to fully distinguish mediation from residual confounding between dietary preference and metabolic outcomes in this observational setting.

While previous studies have shown that genetic variation in taste receptor pathways can influence susceptibility to metabolic disorders [45], our results extend this evidence by highlighting a potential direct metabolic link independent of lifestyle factors. These insights are critical for personalized nutrition, as understanding an individual’s genetic predisposition for the consumption of bioactive-rich foods, such as grapefruit, could help identify individuals whose natural dietary habits support their metabolic health [46,47]. This is particularly relevant since aligning dietary interventions with individual factors is known to enhance long-term adherence and effectiveness [48]. Thus, this research suggests the long-term future perspective of integrating the genetics of dietary behavior into precision nutrition frameworks to optimize metabolic health.

Our molecular docking results provide a plausible mechanistic hypothesis for why *ADCK1* emerged as the key candidate from our GWAS. The finding that naringenin and bergamottin, key drivers of grapefruit taste, can occupy the ATP-binding pocket of ADCK1 suggests that this protein may function as a hypothetical chemosensor for dietary flavonoids [49,50]. This adds a new dimension to its established role in mitochondrial function and coenzyme Q biosynthesis, processes critical for energy metabolism [51]. It posits ADCK1 as a potential molecular link between the intake of a specific food and the modulation of a key metabolic enzyme [52]. Rather than altering classical gustatory taste perception on the tongue, we hypothesize a post-ingestive metabolic feedback loop: if exogenous grapefruit compounds modulate ADCK1’s kinase activity and energy metabolism, this physiological response may subconsciously influence a person’s long-term behavioral preference or aversion to the fruit. This proposed mechanism of speculative competitive inhibition also offers a plausible explanation for the locus’s connection to obesity risk, as such modulation could consequently impact mitochondrial function and energy homeostasis [52]. This biological effect could occur irrespective of the total amount of grapefruit a person consumes. While this hypothesis requires experimental validation through *in vitro* kinase assays to confirm the inhibitory effect of these compounds on the ADCK1 protein, our study suggests a significant connection between a prioritized candidate locus for food consumption and a key metabolic enzyme, opening a new perspective on how food-related genetics and metabolic health are intertwined.

Despite these findings, several limitations warrant consideration. First, grapefruit intake was assessed via self-reported questionnaires, which may involve inherent reporting bias. Second, the study population was predominantly of European ancestry, potentially limiting generalizability to other ethnic groups. Third, as no variants reached the strict genome-wide significance threshold (*p* < 5 × 10^−8^) and independent external validation cohorts were not available, our findings remain suggestive; thus, the possibility of spurious signals cannot be entirely ruled out. Fourth, while we propose a plausible biological mechanism for *ADCK1*, our insights currently rely on computational functional analyses, such as molecular docking simulations, and necessitate further *in vitro* biological validation. Finally, statistical limitations, including the absence of multiple testing correction in secondary analyses and the lack of an explicit test for genotype-by-sex interaction, may increase the risk of Type I errors. Consequently, these hypothesis-generating results require cautious interpretation and must be further replicated in diverse populations before definitive conclusions can be drawn.

Despite these limitations, the primary strength of this study lies in its hypothesis-generating nature. By integrating large-scale genetic data with molecular modeling and detailed phenotypic analysis, our research proposes a prioritized candidate gene, *ADCK1* for grapefruit consumption that lies outside of the canonical taste receptor pathways. We have proposed a plausible molecular mechanism linking a specific dietary behavior directly to a key metabolic enzyme. This work provides a valuable foundation for future experimental studies and contributes a new perspective on the complex interplay between diet, genetics, and metabolic health.

**Figure 4:**
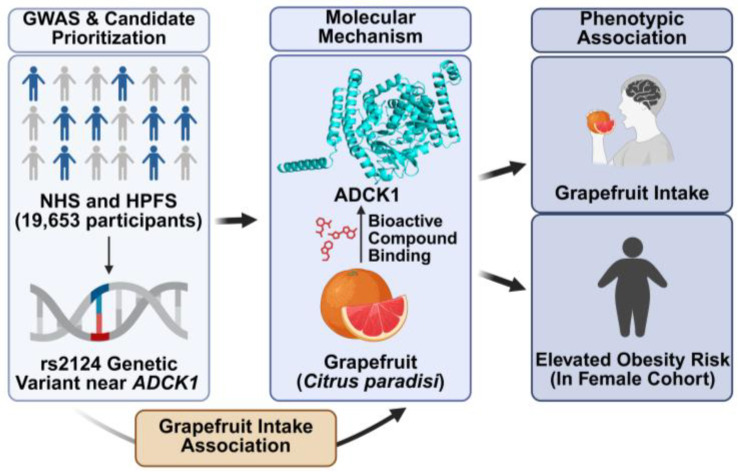
Proposed mechanism linking the *ADCK1* genetic variant rs2124 to grapefruit intake and obesity risk. This exploratory GWAS in the NHS and HPFS cohorts (*n* = 19,653) suggested an association between the rs2124 variant near the *ADCK1* gene and grapefruit intake. The proposed molecular mechanism involves the binding of bioactive compounds from grapefruit (*Citrus paradisi*) to the ADCK1 protein, leading to phenotypic outcomes including differences in grapefruit consumption and an increased risk of obesity observed in the female cohort.

In conclusion, this exploratory study suggested a prioritized candidate locus for grapefruit intake near the metabolic gene *ADCK1*. We also observed a potential cohort-specific link between this locus and obesity risk in females, an association considered a hypothesis-generating signal that operates independently of lifestyle behaviors such as physical activity. The proposed pathway, from the genetic variant to its molecular mechanism and phenotypic outcomes, is summarized in Figure 4. Our findings generate a new hypothesis that genetic factors influencing the consumption of specific foods may act through direct interactions with metabolic pathways, not just classical sensory receptors.

## 5. Conclusions

Taken together, the results of this study suggested prioritized candidate genetic variants associated with grapefruit taste preference and explored their potential independent link to obesity risk through lifestyle and dietary behaviors, as described in Figure 4. Our findings suggest that taste-related genetics may influence long-term metabolic health by shaping individual food choices and dietary patterns. These insights highlight the long-term future perspective of incorporating taste genetics into precision nutrition strategies aimed at improving dietary adherence and reducing obesity risk.

## Figures and Tables

**Table 1:** Lead SNPs associated with grapefruit taste preference suggested by GWAS.

Lead SNPs	Adjacent Gene	CHR	BP	Effect Allele	Non-Effect Allele	MAF	OR (95% CI)	*p*-Value	Q	*I* ^2^	CADD	RDB	eQTL (Tissue)
rs11711528	PRICKLE2	3	64428948	T	C	0.31	0.84 (0.78–0.90)	3.16 × 10^−6^	0.60	0.00	3.44	5	Pancreas, Stomach
rs4975177	RP11-419L4.1	4	130191376	A	G	0.27	1.20 (1.11–1.30)	6.07 × 10^−6^	0.20	26.23	0.98	6	Adipose_Subcutaneous, Adipose_Visceral_Omentum, Esophagus_Mucosa, Pituitary, Cells_Cultured_fibroblasts
rs6555388	CTD-2324F15.2	5	6264666	G	A	0.36	1.22 (1.13–1.32)	1.80 × 10^−7^	0.75	0.00	0.93	7	N/A
rs13236282	OCM	7	5922056	C	T	0.35	0.82 (0.76–0.89)	1.06 × 10^−6^	0.09	40.01	3.82	7	Adipose_Subcutaneous, Esophagus_Gastroesophageal _Junction, Nerve_Tibial
rs12763418	PARD3	10	35075793	C	T	0.23	0.85 (0.80–0.92)	9.42 × 10^−6^	0.06	44.61	6.75	6	Cells_Cultured_Fibroblasts, Skin_Sun_Exposed_Lower_leg, Thyroid, Adrenal_Gland
rs2124	ADCK1	14	78556514	C	T	0.46	0.85 (0.79–0.91)	3.69 × 10^−6^	0.67	0.00	6.61	NA	Brain_Hypothalamus
rs35207503	RBFOX3	17	77325976	T	C	0.05	0.79 (0.71–0.88)	7.82 × 10^−6^	0.90	0.00	3.95	5	N/A
rs72985681	SALL3	18	76761852	T	A	0.18	0.80 (0.73–0.88)	1.01 × 10^−6^	0.27	19.20	4.07	6	Whole Blood

*ADCK1*, aarF domain containing kinase 1; BP, position (hg19); CADD, Combined Annotation Dependent Depletion; CHR, chromosome; CI, confidence interval; eQTL, expression quantitative trait locus; GWAS, genome-wide association study; *I*^2^, heterogeneity; MAF, minor allele frequency; NA, not available; *OCM*, oncomodulin; OR, odds ratio; *PARD3*, par-3 family cell polarity regulator; *PRICKLE2*, prickle planar cell polarity protein 2; Q, Cochran’s Q statistics; RDB, RegulomeDB 2.0; *RBFOX3*, RNA binding fox-1 homolog 3; *RP11-419L4.1*, long non-coding RNA; *CTD-2324F15.2*, RNA, 5S ribosomal 388; *SALL3*, spalt-like transcription factor 3; SNP, single-nucleotide polymorphism.

**Table 2:** Age-standardized characteristics according to genotypes of SNP rs2124 among US males and females in the NHS and HPFS.

	NHS			HPFS		
Genotypes	TT (*n* = 2151)	CT (*n* = 5834)	CC (*n* = 4113)	TT (*n* = 1345)	CT (*n* = 3686)	CC (*n* = 2524)
Age (years)	57 (6.9)	57.2 (6.8)	57.1 (6.9)	57.8 (8.9)	57.6 (8.7)	57.5 (8.6)
Caucasian (%)	99.7	99.7	99.8	95.3	95.0	94.7
BMI (kg/m^2^)	23.5 (2.7)	23.6 (2.7)	23.6 (2.7)	24.8 (2.2)	24.9 (2.2)	24.8 (2.3)
Weight (kg)	63.4 (8.3)	63.8 (8.5)	63.9 (8.5)	79.5 (8.9)	79.4 (9.1)	79.3 (8.8)
Never smokers (%)	43.5	44.6	45.6	48.3	50.8	49.9
Past smokers (%)	39.2	37.2	36.6	44.4	41.7	41.9
Current smokers (%)	16.9	18.0	17.5	7.3	7.5	8.1
Alcohol intake (g/day)	7.7 (10.9)	7.3 (10.7)	7.4 (10.6)	12.6 (16.1)	12.2 (15.4)	12.3 (15.8)
Physical activity (MET-h/week)	15.6 (22.6)	15.2 (20.5)	14.9 (19.4)	20.6 (23.5)	21.2 (24.9)	20.8 (23.7)
Total energy intake (kcal/d)	1765.6 (480.3)	1765.7 (480.4)	1763.5 (481.0)	2005.6 (610.4)	2023.8 (606.8)	2025.6 (594.2)
AHEI	46.2 (9.9)	46.3 (10.0)	45.9 (10.0)	47.0 (10.9)	47.0 (10.9)	46.7 (10.9)
Glycemic load	98.8 (17.1)	98.8 (17.2)	98.5 (17.5)	124.6 (26.4)	124.4 (24.7)	123.5 (25.4)
Trans fat	1.9 (0.6)	1.9 (0.6)	1.9 (0.6)	2.8 (1.2)	2.8 (1.1)	2.9 (1.1)
Total fiber	17.4 (4.7)	17.4 (4.8)	17.3 (4.8)	20.9 (6.7)	21.1 (6.9)	21.0 (6.6)
Fruit	75.7 (44.4)	75.6 (45.2)	73.9 (44.8)	81.0 (203.4)	65.5 (151.3)	69.9 (169.2)
Vegetable	85.8 (45.0)	86.8 (46.8)	84.6 (46.6)	117.7 (324.5)	95.7 (257.9)	99.6 (269.4)
Coffee	18.1 (30.4)	18.4 (30.2)	17.9 (29.4)	25.9 (40.5)	22.8 (34.7)	24.9 (37.5)
Tea	7.0 (6.0)	6.9 (6.2)	6.7 (5.9)	23.7 (37.7)	21.9 (33.7)	23.3 (36.2)
Sweetened beverage	38.2 (40.4)	38.3 (41.4)	37.4 (40.2)	16.8 (26.5)	17.7 (29.3)	17.2 (28.6)
Grapefruit	3.0 (9.9)	2.8 (8.1)	2.6 (8.2)	12.5 (31.6)	11.3 (27.4)	11.6 (28.8)
Grapefruit juice	4.7 (10.8)	4.1 (9.2)	4 (9.3)	37.2 (40.9)	34.7 (36.7)	35.6 (38.8)
Kale	16.6 (13.5)	16.8 (12.9)	16.7 (13.2)	54.0 (28)	51.4 (26.8)	52.9 (28.2)
Sleep (h/day)	7.0 (0.9)	7.0 (0.9)	7.0 (0.9)	7.1 (0.7)	7.1 (0.8)	7.1 (0.8)
Cases/persons-years	359/42,797	1019/113,132	793/79,465	171/24,965	476/69,050	326/47,693
Crude Incidence/100K PY	839	901	998	685	689	684

AHEI, alternate healthy eating index; BMI, body mass index; HPFS, Health Professionals Follow-up Study; MET, metabolic equivalent; NHS, Nurses’ Health Study; PY, person-years. Values are not age-adjusted. Data are presented as mean (SD) for continuous variables and percentages for categorical variables, and are standardized to the age distribution of the study population.

**Table 3:** Adjusted HR of obesity for genotypes of SNPs in the NHS and HPFS.

	Model 1 (Age-Adjusted Model)			Model 2 (Multivariate Adjusted Model)		
Independent Variable	HR	95% CI	*p*-Value	HR	95% CI	*p*-Value
rs2124 (CT vs. TT)						
NHS	1.072	0.950–1.209	0.260	1.065	0.944–1.202	0.304
HPFS	0.995	0.835–1.186	0.958	1.015	0.852–1.210	0.868
Pooled	1.047	0.948–1.156	0.368	1.049	0.950–1.158	0.346
rs2124 (CC vs. TT)						
NHS	1.183	1.044–1.341	0.008	1.177	1.039–1.334	0.011
HPFS	0.978	0.812–1.177	0.814	0.983	0.817–1.184	0.860
Pooled	1.115	1.005–1.237	0.039	1.113	1.003–1.235	0.043
rs713598 (CG vs. CC) ^1^						
NHS	1.132	1.029–1.245	0.011	1.130	1.027–1.243	0.012
HPFS	0.957	0.831–1.102	0.540	0.962	0.835–1.108	0.587
Pooled	1.074	0.992–1.162	0.078	1.074	0.993–1.163	0.076
rs713598 (GG vs. CC)						
NHS	1.155	1.019–1.308	0.024	1.141	1.007–1.293	0.038
HPFS	1.077	0.899–1.290	0.422	1.090	0.910–1.307	0.349
Pooled	1.129	1.019–1.251	0.021	1.125	1.015–1.246	0.025

CI, confidence interval; HPFS, Health Professionals Follow-up Study; HR, hazard ratio; NHS, Nurses’ Health Study; SNP, single-nucleotide polymorphism; Model 1 is age-adjusted; Model 2 is adjusted for age, smoking status, alcohol intake, physical activity, total fiber, trans fat, total energy intake and sweetened beverage intake to control for the confounding effects of lifestyle and added sugar; Follow-up for both the NHS and HPFS was from 1986 to 2014; Results of two cohorts were pooled by means of inverse variance-weighted fixed-effects meta-analysis (all *p*-values for heterogeneity > 0.05). *p*-values are nominal and not corrected for multiple comparisons. ^1^ SNP reported to be associated with bitter taste receptor gene *TAS2R38* in the study by Duffy et al. [43].

## Data Availability

The data used in this study (NHS and HPFS) are managed by the Channing Division of Network Medicine, Brigham and Women’s Hospital and Harvard T.H. Chan School of Public Health. Data are available upon reasonable request through the formal application process at the Channing Division of Network Medicine.
